# Sex and Age Differences in Glucocorticoid Signaling After an Aversive Experience in Mice

**DOI:** 10.3390/cells13242041

**Published:** 2024-12-10

**Authors:** Yun Li, Bin Zhang, Youhua Yang, Ping Su, James Nicholas Samsom, Albert H. C. Wong, Fang Liu

**Affiliations:** 1 Oujiang Laboratory (Zhejiang Lab for Regenerative Medicine, Vision, and Brain Health), Institute of Mental Health and Drug Discovery, School of Mental Health, Wenzhou Medical University, Ouhai District, Wenzhou 325000, China; bingofire@126.com (B.Z.); yangyouhua1229@wmu.edu.cn (Y.Y.); 2 Laboratory of Brain Disorders, Beijing Institute of Brain Disorders, Ministry of Science and Technology, Collaborative Innovation Center for Brain Disorders, Capital Medical University, Beijing 100054, China; lujiuly22321@163.com (Y.L.); suping67@zju.edu.cn (P.S.); 3 Campbell Family Mental Health Research Institute, Centre for Addiction and Mental Health, 250 College St., Toronto, ON M5T 1R8, Canada; james.samsom@camh.ca (J.N.S.); albert.wong@utoronto.ca (A.H.C.W.); 4 Department of Pharmacology & Toxicology, University of Toronto, 250 College St., Toronto, ON M5T 1R8, Canada; 5 Institutes of Medical Science, University of Toronto, 1 King’s College Cir., Toronto, ON M5S 1A8, Canada; 6 Department of Psychiatry, University of Toronto, 250 College St., Toronto, ON M5T 1R8, Canada; 7 Department of Physiology, University of Toronto, 1 King’s College Cir., Toronto, ON M5S 1A8, Canada

**Keywords:** corticosterone, cortisol, glucocorticoid receptor, fear conditioning

## Abstract

Background: glucocorticoids may play an important role in the formation of fear memory, which is relevant to the neurobiology of post-traumatic stress disorder (PTSD). In our previous study, we showed the glucocorticoid receptor (GR) forms a protein complex with FKBP51, which prevents translocation of GR into the nucleus to affect gene expression; this complex is elevated in PTSD patients and by fear-conditioned learning in mice, and disrupting this complex blocks the storage and retrieval of fear-conditioned memories. The timing of release of glucocorticoid relative to the formation of a traumatic memory could be important in this process, and remains poorly understood. Methods and Results: we mapped serum corticosterone over time after fear conditioning in cardiac blood samples from male and female mice, as well as adult and aged mice using ELISA. We show a significant alteration in serum corticosterone after conditioning; notably, levels spike after 30 min but drop lower than unconditioned controls after 24 h. We further investigate the effect of glucocorticoid on GR phosphorylation and localization in HEK 293T cells by Western blot. Hydrocortisone treatment promotes phosphorylation and nuclear translocation of GR. Conclusions: these data contribute to our understanding of the processes linking stress responses to molecular signals and fear memory, which is relevant to understanding the shared mechanisms related to PTSD.

## 1. Introduction

Traumatic events can sometimes result in a syndrome of persistent anxiety, flashbacks, avoidance, and hypervigilance called PTSD (post-traumatic stress disorder). Current treatment consists of selective serotonin reuptake inhibitor antidepressants, such as paroxetine and sertraline, in combination with exposure therapy [1,2,3]. Other treatments such as eye movement desensitization, prazosin for nightmares, and cannabis, are also common, but efficacy for all existing remedies is limited [4,5,6,7]. The hypothalamic–pituitary–adrenal axis (HPA), including cortisol and glucocorticoid receptor (GR), have important functions in response to stress and trauma [8], but much of the underlying neurobiology remains unclear.

One important question about the mechanism of PTSD is why only some people develop symptoms after exposure to a traumatic event: for example, in military units where many soldiers experience almost the same events, while only a few develop PTSD [9]. Prior exposure to trauma, especially in childhood, may increase susceptibility to PTSD after adult trauma [10], possibly through epigenetic modifications that alter transcriptional responses to adult trauma [8,11,12]. Recent research has identified a GR chaperone protein, FKBP51, as having a critical role in this trauma priming phenomenon [13]. We demonstrated that GR and FKBP51 directly bind to form a protein complex that is elevated in patients with PTSD and by fear conditioning in mice [14]. FKBP51 prevents FKBP52-mediated nuclear translocation of GR. Additionally, disruption of the GR-FKBP51 complex blocked the formation of fear-conditioned memories and inhibited fear-conditioned freezing in mice without disrupting spatial, object, or social memory [15]. Thus, blocking the GR-FKBP51 interaction could be a potential treatment for PTSD; however, we lack a detailed understanding of how trauma interacts with the stress response in regulating GR and its effectors.

HPA axis activation and cortisol release are established biological responses to stress, including psychological trauma [10,16,17]. Consequently, there have been many studies examining cortisol levels and other biomarkers related to the HPA axis in PTSD [reviewed [18]]. Unfortunately, there are many contradictory findings, with no clear consensus of increased or decreased cortisol in PTSD patients. These inconsistent findings could be due to limitations inherent in human clinical studies. Cortisol levels fluctuate in a cyclic fashion at baseline, requiring careful timing of samples, and preferably more than one sample to provide reliable data for each subject [19,20]. Just as important is the timing of cortisol sample collection relative to the traumatic event and the patient’s symptom presentation, all of which are very difficult to standardize in a real-world scenario. PTSD is not an endocrine pathology, so differences in cortisol would fall within the range of normal variability [18]. This further complicates human studies, as cortisol can vary based on ordinary patient characteristics such as weight and gender, as well as life experiences. Indeed, studies with more homogeneous patient populations were more likely to find significant differences in cortisol in PTSD [21]. Because of the limitations inherent in human clinical studies, animal and cell model systems are a pragmatic alternative to mapping the trajectory of glucocorticoid release after a traumatic event. Understanding the hormonal environment following a traumatic event will help connect the stress response and process of fear-memory formation with respect to underlying molecular mechanisms such as the activation of GR and the aforementioned GR-FKBP51 complex formation.

The fear-conditioning paradigm represents the simplest model with which to examine the underlying neurobiology that is precipitated by a traumatic experience [reviewed in [22]]. We previously showed that PTSD-related FKBP51 molecular mechanism affects fear conditioning in mice [15]. To further examine the stress response that occurs following a traumatic experience, we measured the time course of the glucocorticoid response to fear learning in mice. Sex and age are known to influence PTSD rates, with females and younger individuals experiencing higher rates [23,24]. We included sex and age as factors in examining the glucocorticoid response to explore potential differences that could underlie sex and age effects on PTSD risk. In addition, to further understand the cellular mechanisms which relate to our previous work on the GR-FKBP51 complex, we also performed hydrocortisone challenge experiments in HEK 293T cells to examine phosphorylation and nuclear translocation of GR in response to glucocorticoids. This knowledge aids in bridging the gap in our understanding of the mechanisms underlying fear memory and PTSD.

## 2. Materials and Methods

### 2.1. Animals

Male and female C57BL/6J mice, aged 6–8 weeks, were purchased from Beijing Vital River Laboratory Animal Technology Co., Ltd. (Beijing, China). through the animal department of Capital Medical University and raised in a specific pathogen-free environment. Mice were acclimatized to the vivarium for 1 week prior to the start of procedures. An additional group of mice was housed in the vivarium until 64-weeks-old, to understand age as a factor. The vivarium consisted of an SPF grade temperature (24  ±  1 °C) and humidity (50 ±  5%) controlled environment (12 h light/dark, 7h00 on). All animal experimental procedures were approved by the Institutional Animal Care and Use Committee of the Capital Medical University, Beijing, China.

### 2.2. Cued Fear Conditioning

For the establishment of fear-conditioned model, animals were first habituated to the apparatus (Med Associates Inc.) for 3 min, then exposed to 30 s of the cue (an in-house white light), which co-terminated with a 1 s foot-shock (0.5 mA) as the conditioned stimulus (CS). Cue exposure CS pairings were repeated 5 times with 5 min inter-trial intervals (Supplementary Figure S1A). On the 3rd day following conditioning, animals were subjected to extinction training, consisting of 5 rounds of 30 s cue exposure with 5 min inter-trial intervals once per day. The expression of fear was assessed 24 h after the last extinction session (5 days post-conditioning). Before the assessment, context cues within the apparatus were changed, and the foot-shock plate was covered. The assessment consisted of a 3 min period of habituation in the absence of the CS, followed by a 3 min presentation of the CS. Freezing behavior was recorded and scored by automated software (SOF-843 Video Freeze, Med Associates Inc., Alpharetta, GA, USA). After confirming that this training method successfully established a conditioned fear response, a test cohort of mice was trained using the day 1 conditioning procedure with control mice receiving cue-only exposure without the CS. Conditioning occurred between the hours of ZT1.5 and ZT3 (ZT0 = lights on, 7h00). Cardiac blood was collected from separate groups of mice sacrificed at 0, 0.5, 1, 2, 4, 8, 12 and 24 h after training (*n* = 4 per group). Mice were exsanguinated using the cardiac blood collection method under isoflurane anesthesia and blood was transferred to EDTA-K2 anticoagulation tubes (BD Vactutainer). Blood harvest was performed between the hours of ZT3.5 and ZT10.5.

### 2.3. ELISA

Whole blood was processed by centrifugation at 1000× *g* for 10 min at room temperature, and the plasma layer was collected. Murine plasma corticosterone and cortisol concentrations were measured with Corticosterone or Cortisol ELISA kits (Abcam, ab108821, Cambridge, England. LifeSpan BioSciences Inc. LS-F10026, Seattle, WA, USA) according to manufacturer instructions. Samples were diluted 100× before measurement. Samples were quantified relative to the standard curve included in each kit.

### 2.4. Cell Culture and Transfection

HEK 293T cells were grown in Dulbecco’s Modified Eagle Medium (DMEM) supplemented with 10% (*v*/*v*) fetal bovine serum (FBS) and 1% penicillin/streptomycin solution (P/S). All cell lines were grown at 37 °C in 5% CO2 atmosphere. Cells were split onto six-well poly-D-lysine coated plates at 60–70% confluency for transfection. Lipofectamine™ 3000 Transfection Reagent (Thermo Fisher Scientific, Waltham, MA, USA) and 3 μg plasmid (FLAG—GR: GFP—FKBP51 = 1: 1) was added, and cells were incubated for 24 h.

### 2.5. Hydrocortisone Administration

In the variable-concentration administration experiments, hydrocortisone (Tocris, Catalog number: 4093) was added to transfected cultures to concentrations of 0.1 nM, 1 nM, 10 nM, 100 nM and 1000 nM. The control group was given an equal volume of the vehicle (DMSO). After 24 h, the cells were collected for protein extraction. In the time-variable experiments, 100 nM hydrocortisone was added to cultures, and the cells were collected for protein extraction after 0 h, 0.5 h, 1 h, 2 h, 4 h, 8 h, 12 h and 24 h. A no-hydrocortisone condition was also included as a control.

### 2.6. Nuclear- and Cytoplasmic-Protein Western Blot Analysis

Nuclear- and cytoplasmic-protein isolation assays were performed in HEK 293T cells using the Subcellular Protein Fractionation Kit (Thermo Fisher Scientific, Waltham, MA, USA) for Cultured Cells according to manufacturer instructions. Whole-cell proteins were isolated with RIPA buffer (Sigma, St. Louis, MO, USA). The same amount of protein (30 μg) from each sample was boiled for 8 min in 5× protein SDS PAGE loading buffer and subjected to 10% SDS-PAGE. After transfer of proteins onto nitrocellulose, membranes were subject to Western blot with the primary antibodies. The protein level was quantified by densitometry (ImageJ v1.52g). The antibodies used included glucocorticoid receptor (GR) (Proteintech, rabbit, catalog 24050-1-AP, Chicago, IL, USA), anti-Histone H3 (Abcam, rabbit, catalog ab1791, Cambridge, England.), anti-phospho-GR (Ser211) (Cell Signaling Technology, rabbit, catalog 4161, Danvers, MA, USA), and anti-α-tubulin (Sigma, mouse, catalog T9026, St. Louis, MO, USA) as a housekeeping control.

### 2.7. Statistical Analysis

The densitometry of protein levels were normalized to the mean value of control levels from the same blot. Results for each sample are presented as the percentage of the control group. For ELISA analyses, technical triplicates were averaged, and 450 nm absorbances were converted to ng/mL by fitting a log–log regression line to the standard curve values. ANOVA assumptions were tested with Shapiro–Wilk’s test for normality and Bartlett’s test for homogeneity of variance, and by examining the residual plots for homoscedasticity and normality of residuals. Boxplots were checked for outliers (>3× interquartile range). Three outliers were removed for the cortisol measurements (conditioned female 4 h, 8 h, and 24 h). No outliers were detected for corticosterone or protein measurements. ANOVAs were used to compare groups with post hoc marginal means comparisons. False discovery rate (fdr) corrections were applied to sets of contrasts for the post hoc analysis. Data were analyzed by ImageJ v1.52g for densitometry and R for statistical modeling.

## 3. Results

### 3.1. Corticosterone Levels After Fear Conditioning in Adult and Aged Male and Female Mice

**Figure 1:**
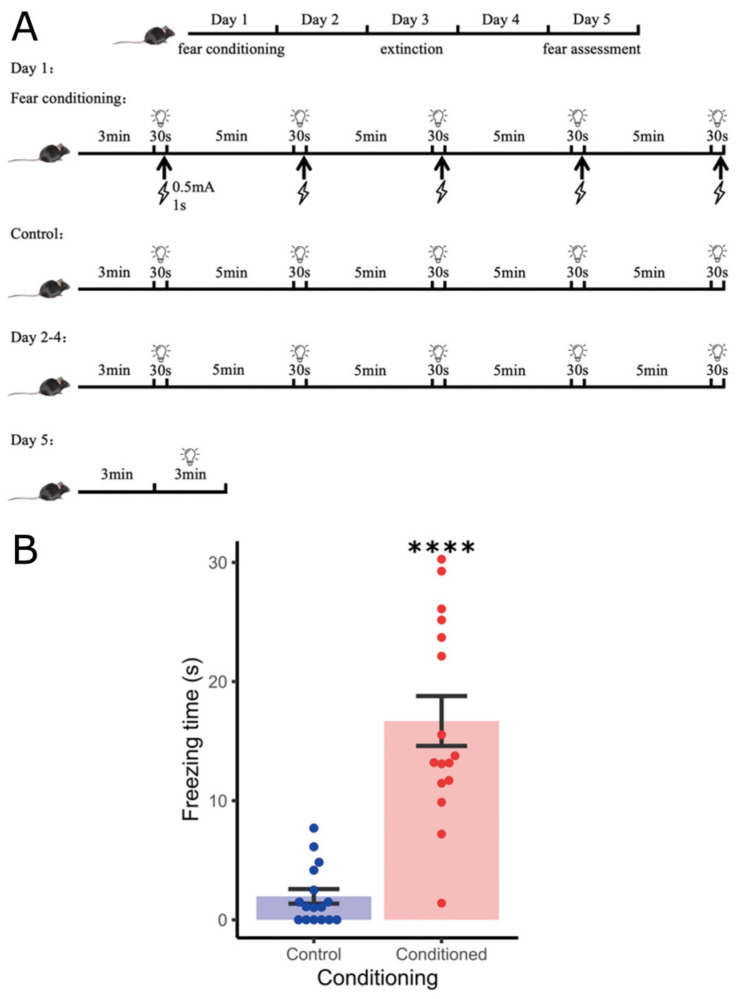
Successful establishment of fear-conditioned mouse model. (**A**) A schematic illustration schedule for fear conditioning. The conditioned stimulus (CS) was a white light illuminated for 30 s, the unconditioned stimulus (US) was a 1 s 0.5 mA foot shock. Conditioned animals received 5 CS-US pairings, control animals received the CS alone. (**B**) Time spent freezing during the 3 min CS presentation on day 5 in adult male (8-week-old) mice. Conditioned animals showed significantly more freezing behavior compared to controls. **** *p* < 0.0001, n = 16, permutation test.

We used fear conditioning in mice as a model to examine how glucocorticoid levels change in response to a stressful learning experience. Firstly, we exposed mice to five light-foot shock pairings and measured the freezing response to the conditioned stimulus after extinction training to validate if our procedure caused robust fear-memory formation (Figure 1A). Conditioned mice spent significantly more time freezing (*p* < 0.00001), averaging 14.7 s [95CI: 10.6; 18.9] longer freezing times compared to controls, demonstrating effective fear memory even at 5 days post conditioning, with extinction training (Figure 1B).

**Figure 2:**
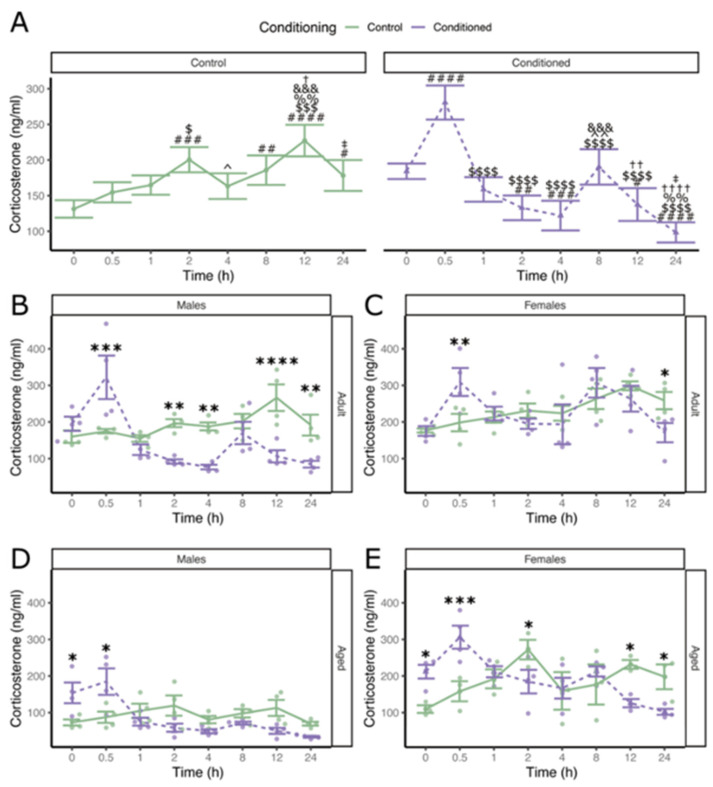
Fear conditioning affects the time course of corticosterone levels in mice. (**A**) Corticosterone protein-expression response curves over 24 h in unconditioned (Control) and fear-conditioned animals (Conditioned). Comparison of corticosterone levels over time in conditioned (dashed) and unconditioned (solid) adult (8-week-old) male (**B**), adult female (**C**), aged (64-week-old) male (**D**), and aged female (**E**) mice. Data are shown as mean ± SEM, 4-way ANOVA (n = 4 *, female aged control n = 3), -corrected post hoc marginal means. Significance indicators: ^#^ *p* < 0.05, ^##^ *p* < 0.01, ^###^ *p* < 0.001, ^####^ *p* < 0.0001 relative to 0 h; ^$^ *p* < 0.05, ^$$$^ *p* < 0.001, ^$$$$^ *p* < 0.0001 relative to 0.5 h; ^%%^ *p* < 0.01 relative to 1 h; ^^^ *p* < 0.05, ^^^^ *p* < 0.01 relative to 2 h, ^&&&^ *p* < 0.001 relative to 4 h, ^†^ *p* < 0.05, ^††^ *p* < 0.005, ^††††^ *p* < 0.0001 relative to 8 h; ^‡^ *p* < 0.05 relative to 12 h. * Control relative to Conditioned; * *p* < 0.05, ** *p* < 0.01, *** *p* < 0.001, **** *p* < 0.0001.

We next subjected a new cohort of mice to the conditioning procedure and sacrificed them 0.5, 1, 2, 4, 8, 12 and 24 h after the final CS (light)-foot shock pairing (controls received the CS alone, with no shock). We measured plasma corticosterone levels in cardiac blood samples from separate groups of male and female adult (8-week-old) or aged (64-week-old) mice. There was a significant interaction between conditioning and time (F_7,183_ = 19.38, *p* < 0.00001, η_p_^2^ = 0.43 [0.33; 1]). Post hoc contrasts showed corticosterone levels increased over time in controls (0–24, t(183) = −2.87, *p* = 0.011, d = −1.06 [−1.79; −0.32]) (see Figure 2A for all significant contrasts).

Contrastingly, after conditioning there was a notable peak in corticosterone after half an hour (0–139 0.5 h, t(183) = −5.82, *p* < 0.00001, d = −2.06 [−2.79; −1.33]; 0.5–1 h, t(183) = 7.36, *p* < 0.00001, 140 d = 2.60 [1.86; 3.35]). This demonstrates the significant effect of an aversive experience on the time course of plasma corticosterone over 24 h.

There were significant interactions between conditioning and sex (F_1,183_ = 5.09, p = 0.025, η_p_^2^ = 0.03 [0; 1]), sex and time (F_7,183_ = 2.70, *p* = 0.011, η_p_^2^ = 0.09 [0.01; 1]), sex and age (F_1,183_ = 11.20, *p* = 0.00099, η_p_^2^ = 0.06 [0.02; 1]), and age and time (F_7,183_ = 3.011, *p* = 0.0051, η_p_^2^ 149 = 0.10 [0.02; 1]) with respect to corticosterone levels. All groups had significantly elevated serum corticosterone in conditioned animals vs. controls 30 min after conditioning (adult male, t(183) = −4.45, *p* = 0.00024, d = −3.14 [−4.58; −151; 1.71]; adult female, t(183) = −3.35, *p* = 0.0063, d = −2.37 [−3.35; −0.0063]; aged male, t(183) = −152; 2.93, *p* = 0.015, d = −2.07 [−3.49; −0.66]; aged female, t(183) = −4.13, *p* = 0.00058, d = −3.16 [−153; 4.70; −1.61]) (Figure 2B–E).

**Figure 3:**
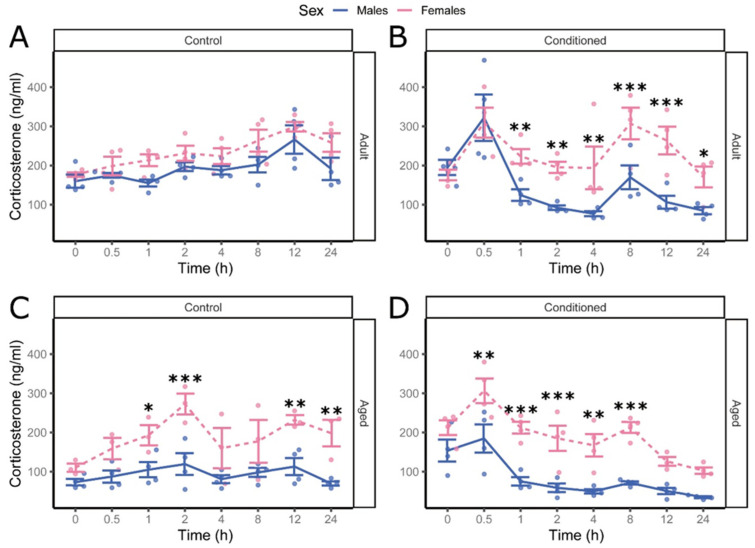
Female mice have increased corticosterone over time, relative to males, after fear conditioning. Comparison of corticosterone levels over time in male (solid) and female (dashed) adult (8-week-old) unconditioned control mice (**A**), adult conditioned (**B**), aged (64-week-old) control (**C**), and aged conditioned (**D**) mice. Data are shown as mean ± SEM, 4-way ANOVA (n = 4 *, female aged control n = 3), fdr-corrected post hoc marginal means, * *p* < 0.05, ** *p* < 0.01, *** *p* < 0.001.

Corticosterone levels in adult male and female control mice did not differ over the measurement period (Figure 3A), while female conditioned mice had significantly higher levels than males at later time points following conditioning (1 h, t(183) = −3.11, *p* = 0.0058, d = −2.11 [−3.52; −0.70]); 8 h, 167 t(183) = −4.15, *p* = 0.00035, d = −2.93 [−4.36; −1.50]; 12 h, t(183) = −4.75, *p* = 0.00013, d = −3.36; 168 [−4.80; −1.92]; 24 h, t(183) = −2.60, *p* = 0.023, d = −1.84 [−3.25; −0.43]) (Figure 3B). Aged male mice had significantly lower corticosterone than females in both control (2 h, t(183) = −4.29, *p* = 0.00033, d = −3.27 [−4.82; −1.73]; 12 h, t(183) = −3.34, *p* = 0.0030, d = −2.55 [−4.08; −1.02]; 24 h, t(183) = −3.59, *p* = 0.0016, d = −2.74 [−4.27; −1.21]) and conditioned groups (0.5 h, t(183) = −172 3.68, *p* = 0.0014, d = −2.60 [−4.03; −1.18]; 2 h, t(183) = −3.82, *p* = 0.00098, d = −2.70 [−4.12; −173; 1.28]; 8 h, t(183) = −4.27, *p* = 0.00033, d = −3.02 [−4.45; −1.59]) (Figure 3C,D). This suggests that aged male mice have decreased corticosterone levels compared to adult males, and younger females maintained higher levels after the initial spike following conditioning, compared to males.

**Figure 4:**
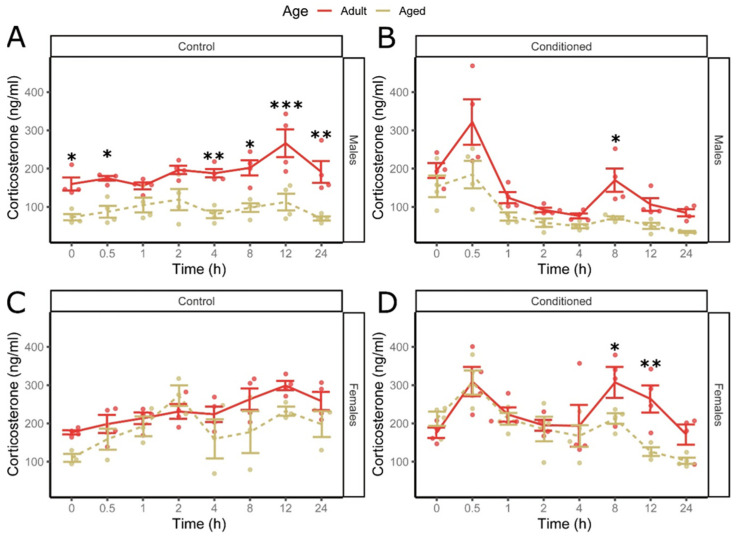
Age reduces corticosterone levels and reactivity to fear conditioning. Comparison of corticosterone levels over time in adult (8-week-old) (solid) and aged (64-week-old) (dashed) male unconditioned control mice (**A**), male conditioned (**B**), female control (**C**), and female conditioned (**D**) mice. Data are shown as mean ± SEM, 4-way ANOVA (n = 4 *, female aged control n = 3), fdr-corrected post hoc marginal means, * *p* < 0.05, ** *p* < 0.01, *** *p* < 0.001.

Male controls had significantly lower corticosterone as they aged (0 h, t(183) = 2.62, *p* = 0.030, d = 1.85 [0.44; 3.26]; 4 h, t(183) = 3.23, *p* = 0.0093, d = 2.29 [0.87; 3.70]; 12 h, t(183) = 4.64, *p* = 0.00021, d = 3.28 [1.84; 4.71]; 24 h, t(183) = 3.67, *p* = 0.0026, d = 2.59 [1.17; 4.01]) (Figure 4A). Aged males reacted broadly the same to conditioning as young males, except for significantly lower levels after 8 h (8 h, t(183) = 2.97, *p* = 0.015, d = 2.10 [0.69; 3.51]) (Figure 4B). Control aged females did not differ from their younger counterparts (Figure 4C), but after conditioning, aged females also had significantly lower levels after 8 h (8 h, t(183) = 2.85, *p* = 183 0.020, d = 2.01 [0.60; 3.42]; 12 h, t(183) = 3.84, *p* = 0.0018, d = 2.93 [1.40; 4.47]) (Figure 4D). This suggests aged mice had a blunted corticosterone response.

### 3.2. Cortisol Levels After Fear Conditioning in Male and Female Mice

Corticosterone is the main glucocorticoid produced in laboratory rodents; however, they also produce cortisol, which is the major stress hormone in humans and most mammals. We also measured the time course of cortisol levels after conditioning. The time course of cortisol levels in conditioned and control animals appeared visually similar to the corticosterone results, with higher variance (Supplementary Figure S1).

### 3.3. Hydrocortisone Regulates GR Phosphorylation and Subcellular Localization in a Concentration-Dependent Manner

**Figure 5:**
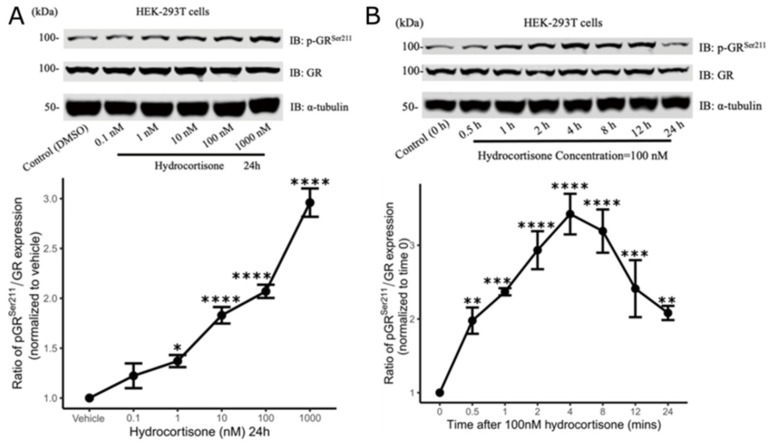

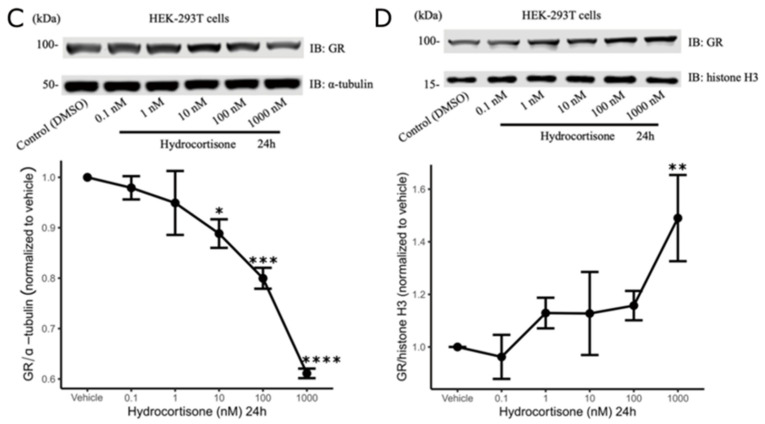
Hydrocortisone treatment stimulates Ser211 phosphorylation of glucocorticoid receptor (GR) and promotes nuclear translocation of GR in HEK 293T cells. (**A**) Representative Western blots (**top**) and densitometric analysis (**bottom**) of phosphorylated GR-S211 (pGR^Ser211^) in HEK 293T whole-cell lysates stimulated by different concentrations of hydrocortisone. The level of pGR^Ser211^ is expressed as a ratio relative to levels of unphosphorylated GR normalized to the vehicle (DMSO) alone condition. (**B**) Representative Western blots (**top**) and densitometric analysis (**bottom**) of the levels of pGR^Ser211^ in HEK 293T cells stimulated by hydrocortisone (100 nM) over time. pGR^Ser211^/GR ratios were normalized to time 0. (**C**) Representative Western blots (**top**) and densitometric analysis (**bottom**) of the changes in cytoplasmic GR protein expression in HEK 293T cells stimulated by different concentrations of hydrocortisone. GR levels expressed relative to α–tubulin and normalized to vehicle (DMSO). (**D**) Representative Western blots (**top**) and densitometric analysis (**bottom**) of the changes in nuclear GR protein expression in HEK 293T cells stimulated by different concentrations of hydrocortisone. GR levels expressed relative to histone H3 and normalized to vehicle (DMSO). Data are shown as mean ± SEM, one-way ANOVA (n = 3), * *p* < 0.05, ** *p* < 0.01, *** *p* < 0.001, **** *p* < 0.0001.

Glucocorticoids exert their effects on the stress response and other physiological effects through binding to the ligand-dependent transcription factor, GR, to initiate downstream gene regulation [25]. We previously showed FKBP51 is important for regulating GR [14], so we examined the effects of cortisol (hydrocortisone) in a HEK 293 cell system expressing FKBP51. GR phosphorylation and subcellular localization are both fundamental aspects of GR function [26,27]. For instance, phosphorylation aids GR sumoylation and cross talk in the JNK and SUMO pathways, which fine-tune GR transcriptional activity in a target gene-specific manner, thereby modulating the hormonal response of cells exposed to stress [28,29]. We first measured GR phosphorylation at serine (Ser211) in response to hydrocortisone treatment in whole-cell lysates from HEK293 cells using Western blots. Hydrocortisone increased GR Ser211 phosphorylation (pGR^Ser211^) in a concentration-dependent manner (F_5,12_ = 60.66, *p* < 0.0001, η^2^ = 0.96) (Figure 5A). After 100 nM hydrocortisone treatment, GR phosphorylation levels rose significantly (F_7,16_ = 11.38, p < 0.0001, η^2^ = 0.83), peaking at 4 h and not quite falling to baseline at the 24 h mark (Figure 5B). We next used Western blots to measure GR expression in different cell compartment fractions from HEK293 cells to examine nuclear translocation response 24 h after hydrocortisone treatment. Hydrocortisone facilitated nuclear localization of GR. Cytoplasmic GR levels decreased with increasing hydrocortisone dose (F_5,12_ = 21.93, *p* < 0.0001, η^2^ = 0.90) (Figure 5C). Conversely, nuclear GR expression increased (F_5,12_ = 3.20, *p* = 0.046, η^2^ = 0.57), although the measured effect was weaker than in the cytosol (Figure 5D).

## 4. Discussion

Our results show a transient spike of corticosterone immediately after exposure to aversive stress in the form of a foot shock in mice. There is also a suppression of corticosterone the day after foot shock, except in aged males, where this did not reach significance. The lack of significance in this group may be due to lower levels overall in the aged mice making it more difficult to measure further reductions. The shape of the corticosterone response curve was the same in adult and aged male mice, which supports this interpretation. Furthermore, next-day suppression in levels was detected in aged females, which had overall higher corticosterone levels. Therefore, it is unlikely that our results reveal a relevant age-related difference in glucocorticoid response. Age and sex appeared to affect levels across the entire experiment, while the fear experience during conditioning affected the pattern of levels over time, rather than increasing stress hormones generally. These observations reinforce the difficulty in interpreting clinical data about cortisol levels in PTSD patients, which could be obtained at various points in this trajectory and differ between subjects [19,20]. These results also provide a basis for further experiments to determine the effect of glucocorticoids on the GR-FKBP51 complex and other molecular pathways regulating fear-memory encoding, recall, and PTSD. Similarly, the effect of GR activation and localization is a core component of the stress response and glucocorticoid function [27,30]. Our experiment connects hydrocortisone stimulation of GR to this process.

The control animals in this study underwent sham conditioning to account for the potential stress involved in handling and the novelty of the conditioning apparatus. Handling and novelty stress could explain the increase in corticosterone over time in the control group, as previous studies have found changes in corticosterone even with routine procedures, such as transporting mice on a metal cart, which persist throughout the circadian cycle [31]. A weakness of our method is that we did not have an “unhandled” control to account for baseline variations in glucocorticoid levels. Corticosterone levels are known to vary along a circadian pattern which peaks daily just before the day/night transition (ZT12 for a 12 h light/dark facility), with levels beginning to rise more sharply around mid-day (~ZT6) [32,33,34,35,36]. In the current study, the 8 h and 12 h samples were harvested closest to the circadian peak (8 h = ZT10.5, 12 h = ZT14). Corticosterone levels in previous studies remained higher in the night period after the peak and rose faster during the day, so the peak at 12 h in our data likely reflects the expected circadian fluctuation [31,32,33]. Interestingly, conditioning appears to have enhanced this peak. Some groups have found circadian fluctuations in glucocorticoid may affect amygdala function and fear-memory formation [37,38]. For instance, fear conditioning was less efficient in mice when training occurred just after the day/night transition, and conditioning efficiency during this period could be enhanced by treatment with the GR inhibitor metyrapone [37]. Additionally, fear-extinction training in rats was more effective at ZT16 relative to ZT4, and these differences were affected by viral manipulation of circadian clock genes [38]. Our experiments would need to be repeated with conditioning at different time points in the circadian cycle, to understand the potential consequences of these effects.

The timing in the spikes of stress hormone levels after an aversive experience may have implications for the normal process of memory formation. Hydrocortisone treatments before learning were shown to enhance long-term recall of emotionally arousing images [39]. Stress-induced increases in cortisol were associated with gains in working-memory encoding and maintenance [40]. Cortisol and corticosterone treatments were also shown to modulate reconsolidation, causing enhancement or suppression of memory, depending on the timing and nature of treatment [41]. Suppressing cortisol spikes elicited by re-activation of memories with metyrapone also affects memory re-consolidation [42]. Given that cortisol appears to affect memory in humans, the fluctuations in corticosterone we measured post conditioning may be important for fear-memory consolidation. The normal time course of glucocorticoid activity could be modulated, for example, by using optogenetics of timed infusion of GR inhibitors/agonists, to elucidate the importance of time-specific changes in glucocorticoids in the formation and consolidation of fear memories.

Our cellular experiments suggest that glucocorticoid stimulates phosphorylation and nuclear translocation of GR. We can therefore hypothesize that the altered trajectory of corticosterone release after fear conditioning will increase nuclear translocation of GR in the short term during the initial spike in levels, but this process may be suppressed later on, when levels are shown to be below the controls after 12 and 24 h. In our previous work, we found that among trauma-exposed subjects, only those with PTSD had elevated GR-FKBP51 complex levels, which also has consequences regarding the nuclear translocation of GR [14]. It is unclear how baseline cortisol levels interact with cortisol release after a traumatic event, and how this might affect GR-FKBP51 complex levels. Future experiments could examine this mechanism by using a gradation of foot-shock exposure, including multiple sessions with variable timing, while measuring cortisol release and GR-FKBP51 complex levels, in conjunction with directly controlling cortisol release without fear-conditioned learning. Understanding these processes could greatly improve clinical treatments. Current front-line pharmacological treatments for PTSD act on broad mechanisms, such as modulation of serotonin, dopamine, or GABA neurotransmission, which are not isolated with respect to the expression or formation of distressing memories [43]. A pathway to better treatments would act on the specific mechanisms by which fear memories, rather than other episodic memories, are consolidated and expressed. It could also be fruitful to extend the scope of these types of experiments to mutant mice, which learn fear-memory associations more or less readily, and to perform longer-term experiments that combine early developmental insults with genetic mutations and prolonged assessments after the simulated traumatic event.

This study has several limitations. Our experiments rely on animal and cellular systems that do not attempt to model the unique psychological complexities of PTSD. This limits the scope of our experiments strictly to the mechanisms of fear memory, which must be related to PTSD, via the implication of a shared set of underlying cellular mechanisms. Furthermore, all blood harvesting procedures will potentiate some stress response [44]. Even acute isoflurane was shown to elevate corticosterone levels in female but not male rats [45], which may have influenced the sex differences we measured, although it would not explain the sex × conditioning interaction. Glucocorticoid levels are likely elevated across all samples, due to the blood drawing procedure, though this is unavoidable. Finally, our age groups represent early post-puberty and old age, respectively. The glucocorticoid time course would need to be measured in adolescent (~3-week-old) mice, to understand differences in response relevant to childhood.

## 5. Conclusions

To conclude, we show that the time course of corticosterone and cortisol expression is altered following fear conditioning in mice. We further show that hydrocortisone promotes phosphorylation and nuclear translocation of glucocorticoid receptor in vitro. These findings have implications for understanding the role of glucocorticoids in the processing of fear memories, as well as contextualizing the complex relationship between cortisol and aversive experiences. Understanding these processes is important for understanding the relationship between mechanisms underlying fear memory, which are relevant for PTSD.

## Data Availability

All data supporting the findings of this study are available within the paper and its Supplementary Information.

## Supplementary Materials

The following supporting information can be downloaded at: https://www.mdpi.com/article/10.3390/cells13242041/s1, Figure S1: Conditioning affects the time course of cortisol levels in male and female mice.

## References

[1] Bisson J.I., Roberts N.P., Andrew M., Cooper R., Lewis C. (2013). Psychological therapies for chronic post-traumatic stress disorder (PTSD) in adults. Cochrane Database Syst. Rev..

[2] Hetrick S.E., Purcell R., Garner B., Parslow R. (2010). Combined pharmacotherapy and psychological therapies for post traumatic stress disorder (PTSD). Cochrane Database Syst. Rev..

[3] Jonas D.E., Cusack K., Forneris C.A., Wilkins T.M., Sonis J., Middleton J.C., Feltner C., Meredith D., Cavanaugh J., Brownley K.A. (2013). Psychological and Pharmacological Treatments for Adults with Post-traumatic Stress Disorder (PTSD).

[4] Murdoch M., Sayer N.A., Spoont M.R., Rosenheck R., Noorbaloochi S., Griffin J.M., Arbisi P.A., Hagel E.M. (2011). Long-term outcomes of disability benefits in US veterans with posttraumatic stress disorder. Arch. Gen. Psychiatry.

[5] Raskind M.A., Peskind E.R., Chow B., Harris C., Davis-Karim A., Holmes H.A., Hart K.L., McFall M., Mellman T.A., Reist C. (2018). Trial of Prazosin for Post-Traumatic Stress Disorder in Military Veterans. N. Engl. J. Med..

[6] Shalev A.Y. (2009). Posttraumatic Stress Disorder and Stress-Related Disorders. Psychiatr. Clin. N. Am..

[7] Zatzick D., Jurkovich G.J., Rivara F.P., Wang J., Fan M.-Y., Joesch J., Mackenzie E. (2008). A national US study of posttraumatic stress disorder, depression, and work and functional outcomes after hospitalization for traumatic injury. Ann. Surg..

[8] Menke A., Lehrieder D., Fietz J., Leistner C., Wurst C., Stonawski S., Reitz J., Lechner K., Busch Y., Weber H. (2018). Childhood trauma dependent anxious depression sensitizes HPA axis function. Psychoneuroendocrinology.

[9] Smith T.C., Wingard D.L., Ryan M.A.K., Kritz-Silverstein D., Slymen D.J., Sallis J.F., for the Millennium Cohort Study Team (2009). PTSD prevalence, associated exposures, and functional health outcomes in a large, population-based military cohort. Public Health Rep..

[10] Binder E.B., Bradley R.G., Liu W., Epstein M.P., Deveau T.C., Mercer K.B., Tang Y., Gillespie C.F., Heim C.M., Nemeroff C.B. (2008). Association of FKBP5 polymorphisms and childhood abuse with risk of posttraumatic stress disorder symptoms in adults. JAMA.

[11] Klengel T., Mehta D., Anacker C., Rex-Haffner M., Pruessner J.C., Pariante C.M., Pace T.W.W., Mercer K.B., Mayberg H.S., Bradley B. (2013). Allele-specific FKBP5 DNA demethylation mediates gene-childhood trauma interactions. Nat. Neurosci..

[12] Heim C., Newport D.J., Mletzko T., Miller A.H., Nemeroff C.B. (2008). The link between childhood trauma and depression: Insights from HPA axis studies in humans. Psychoneuroendocrinology.

[13] Binder E.B. (2009). The role of FKBP5, a co-chaperone of the glucocorticoid receptor in the pathogenesis and therapy of affective and anxiety disorders. Psychoneuroendocrinology.

[14] Li H., Su P., Lai T.K., Jiang A., Liu J., Zhai D., Campbell C.T., Lee F.H., Yong W., Pasricha S. (2020). The glucocorticoid receptor-FKBP51 complex contributes to fear conditioning and posttraumatic stress disorder. J. Clin. Investig..

[15] Jiang A., Zhou C., Samsom J., Yan S., Yu D.Z., Jia Z.-P., Wong A.H.C., Liu F. (2022). The GR-FKBP51 interaction modulates fear memory but not spatial or recognition memory. Prog. Neuropsychopharmacol. Biol. Psychiatry.

[16] Dunlop B.W., Wong A. (2019). The hypothalamic-pituitary-adrenal axis in PTSD: Pathophysiology and treatment interventions. Prog. Neuro-Psychopharmacol. Biol. Psychiatry.

[17] Almeida F.B., Pinna G., Barros H.M.T. (2021). The Role of HPA Axis and Allopregnanolone on the Neurobiology of Major Depressive Disorders and PTSD. Int. J. Mol. Sci..

[18] Yehuda R. (2006). Advances in understanding neuroendocrine alterations in PTSD and their therapeutic implications. Ann. N. Y. Acad. Sci..

[19] Maes M., Lin A., Bonaccorso S., Hunsel F., Gastel A.V., Delmeire L., Biondi M., Bosmans E., Kenis G., Scharpé S. (1998). Increased 24-hour urinary cortisol excretion in patients with post-traumatic stress disorder and patients with major depression, but not in patients with fibromyalgia. Acta Psychiatr. Scand..

[20] Yehuda R., Teicher M.H., Trestman R.L., Levengood R.A., Siever L.J. (1996). Cortisol regulation in posttraumatic stress disorder and major depression: A chronobiological analysis. Biol. Psychiatry.

[21] Yehuda R. (2002). Current status of cortisol findings in post-traumatic stress disorder. Psychiatr. Clin. N. Am..

[22] Ross D.A., Arbuckle M.R., Travis M.J., Dwyer J.B., van Schalkwyk G.I., Ressler K.J. (2017). An Integrated Neuroscience Perspective on Formulation and Treatment Planning for Posttraumatic Stress Disorder: An Educational Review. JAMA Psychiatry.

[23] McGinty G., Fox R., Ben-Ezra M., Cloitre M., Karatzias T., Shevlin M., Hyland P. (2021). Sex and age differences in ICD-11 PTSD and complex PTSD: An analysis of four general population samples. Eur. Psychiatry.

[24] Brewin C.R., Andrews B., Valentine J.D. (2000). Meta-analysis of risk factors for posttraumatic stress disorder in trauma-exposed adults. J. Consult. Clin. Psychol..

[25] Oakley R.H., Cidlowski J.A. (2013). The biology of the glucocorticoid receptor: New signaling mechanisms in health and disease. J. Allergy Clin. Immunol..

[26] Wang Z., Frederick J., Garabedian M.J. (2002). Deciphering the phosphorylation “code” of the glucocorticoid receptor in vivo. J. Biol. Chem..

[27] Grad I., Picard D. (2007). The glucocorticoid responses are shaped by molecular chaperones. Mol. Cell. Endocrinol..

[28] Davies L., Karthikeyan N., Lynch J.T., Sial E.-A., Gkourtsa A., Demonacos C., Krstic-Demonacos M. (2008). Cross Talk of Signaling Pathways in the Regulation of the Glucocorticoid Receptor Function. Mol. Endocrinol..

[29] Galliher-Beckley A.J., Cidlowski J.A. (2009). Emerging roles of glucocorticoid receptor phosphorylation in modulating glucocorticoid hormone action in health and disease. IUBMB Life.

[30] Finsterwald C., Alberini C.M. (2014). Stress and glucocorticoid receptor-dependent mechanisms in long-term memory: From adaptive responses to psychopathologies. Neurobiol. Learn. Mem..

[31] Cordingley J.R., Nemzek J., Qi N. (2024). Noise and Vibration Generation and Response of Mice (*Mus musculus*) to Routine Intrafacility Transportation Methods. J. Am. Assoc. Lab. Anim. Sci..

[32] Barriga C., Martín M.I., Tabla R., Ortega E., Rodríguez A.B. (2001). Circadian rhythm of melatonin, corticosterone and phagocytosis: Effect of stress. J. Pineal Res..

[33] Dalm S., Karssen A.M., Meijer O.C., Belanoff J.K., de Kloet E.R. (2019). Resetting the Stress System with a Mifepristone Challenge. Cell. Mol. Neurobiol..

[34] Kakihana R., Moore J.A. (1976). Circadian rhythm of corticosterone in mice: The effect of chronic consumption of alcohol. Psychopharmacologia.

[35] Malisch J.L., Breuner C.W., Gomes F.R., Chappell M.A., Garland T. (2008). Circadian pattern of total and free corticosterone concentrations, corticosteroid-binding globulin, and physical activity in mice selectively bred for high voluntary wheel-running behavior. Gen. Comp. Endocrinol..

[36] Kamakura R., Kovalainen M., Leppäluoto J., Herzig K.-H., Mäkelä K.A. (2016). The effects of group and single housing and automated animal monitoring on urinary corticosterone levels in male C57BL/6 mice. Physiol. Rep..

[37] den Boon F.S., de Vries T., Baelde M., Joëls M., Karst H. (2019). Circadian and Ultradian Variations in Corticosterone Level Influence Functioning of the Male Mouse Basolateral Amygdala. Endocrinology.

[38] Woodruff E.R., Chun L.E., Hinds L.R., Varra N.M., Tirado D., Morton S.J., McClung C.A., Spencer R.L. (2018). Coordination between Prefrontal Cortex Clock Gene Expression and Corticosterone Contributes to Enhanced Conditioned Fear Extinction Recall. eNeuro.

[39] Buchanan T.W., Lovallo W.R. (2001). Enhanced memory for emotional material following stress-level cortisol treatment in humans. Psychoneuroendocrinology.

[40] Stauble M.R., Thompson L.A., Morgan G. (2013). Increases in cortisol are positively associated with gains in encoding and maintenance working memory performance in young men. Stress.

[41] Drexler S.M., Wolf O.T. (2017). The role of glucocorticoids in emotional memory reconsolidation. Neurobiol. Learn. Mem..

[42] Antypa D., Perrault A.A., Vuilleumier P., Schwartz S., Rimmele U. (2021). Suppressing the Morning Cortisol Rise After Memory Reactivation at 4 A.M. enhances Episodic Memory Reconsolidation in Humans. J. Neurosci..

[43] Mansour M., Joseph G.R., Joy G.K., Khanal S., Dasireddy R.R., Menon A., Mason I.B., Kataria J., Patel T., Modi S. (2023). Post-traumatic Stress Disorder: A Narrative Review of Pharmacological and Psychotherapeutic Interventions. Cureus.

[44] Arnold M., Langhans W. (2010). Effects of anesthesia and blood sampling techniques on plasma metabolites and corticosterone in the rat. Physiol. Behav..

[45] Bekhbat M., Merrill L., Kelly S.D., Lee V.K., Neigh G.N. (2016). Brief anesthesia by isoflurane alters plasma corticosterone levels distinctly in male and female rats: Implications for tissue collection methods. Behav. Brain Res..

