# Supplementary material for: Sex and Age Differences in Glucocorticoid Signaling After an Aversive Experience in Mice
Source: Cells. 2024 Dec 10;13(24):2041. doi: 10.3390/cells13242041 (PMC11674875; doi:10.3390/cells13242041)
Supplement: Supplementary file 1 [file cells-13-02041-s001.zip › cells-3324225-supplementary.pdf]

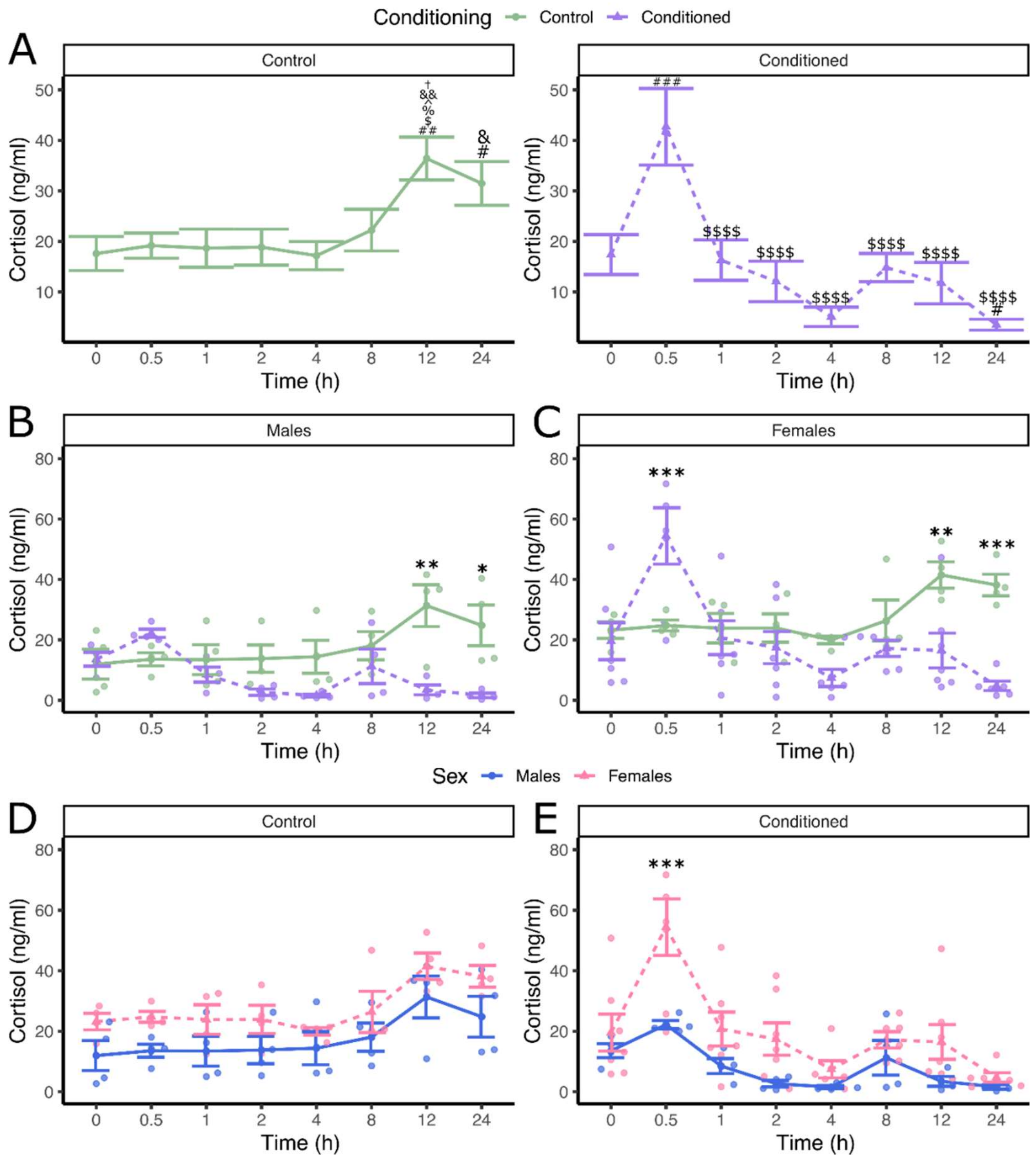

Figure S1. Conditioning affects the time course of cortisol levels in male and female mice. Comparison of cortisol levels over time in adult (8-week-old) conditioned (dashed) or un-conditioned (solid) male (A), female (B) mice. Levels over time in male (solid) and female (dashed) un-conditioned (C), and conditioned (D) mice. Data show mean ± SEM, 3-way ANOVA (n = 4* female conditioned n = 7; conditioning, $F_{1,117} = 20.85$, $p < 0.00001$****, $\eta_p^2 = 0.15$ [0.06, 1]; time, $F_{7,117} = 6.56$, $p < 0.00001$****, $\eta_p^2 = 0.28$ [0.14; 1]; sex, $F_{1,117} = 29.88$, $p < 0.00001$****, $\eta_p^2 = 0.20$ [0.11; 1]; time × conditioning, $F_{7,117} = 8.56$, $p < 0.00001$****, $\eta_p^2 = 0.34$ [0.20; 1]; time × sex, $F_{7,117} = 1.78$, $p = 0.097$, $\eta_p^2 = 0.10$ [0; 1]; sex × conditioning, $F_{1,117} = 0.20$, $p = 0.66$, $\eta_p^2 = 0.0017$ [0; 1]; time × sex × conditioning, $F_{7,117} = 0.76$, $p = 0.62$, $\eta_p^2 = 0.04$ [0; 1]), fdr corrected *post hoc* marginal means. Significance indicators: $^{\#}p < 0.05$, $^{\#\#}p < 0.01$, $^{\#\#\#}p < 0.001$ relative to 0 h; $^{\$}p < 0.05$ relative to 0.5 h; $^{\%}p < 0.05$ relative to 1 h; $^{\wedge}p < 0.05$ relative to 2 h, $^{\&\&}p < 0.01$ relative to 4 h, $^{+}p < 0.05$ relative to 8 h. *Control relative to Conditioned; *p < 0.05, **p < 0.01, ***p < 0.001.
